# Implementation of vertical clinical pharmacist service on venous thromboembolism prophylaxis in hospitalized medical patients

**DOI:** 10.1590/S1679-45082014AO2526

**Published:** 2014

**Authors:** Celina Setsuko Haga, Cassio Massashi Mancio, Micheline da Costa Pioner, Fabricia Aparecida de Lima Alves, Andreia Ramos Lira, João Severino da Silva, Fábio Teixeira Ferracini, Wladimir Mendes Borges, João Carlos de Campos Guerra, Claudia Regina Laselva

**Affiliations:** 1 Hospital Israelita Albert Einstein, São Paulo, SP, Brazil

**Keywords:** Venous thromboembolism, Pharmacy service, hospital, Patient safety

## Abstract

**Objective:**

To describe the vertical clinical pharmacist service's interventions in prevention of venous thromboembolism.

**Methods:**

This prospective study was done at a private hospital. From January to May 2012, the clinical pharmacist evaluated medical patients without prophylaxis for thromboembolism. If the patient fulfilled criteria for thromboembolism and did not have contraindications, the clinical pharmacist suggested inclusion of pharmacologic agents and/or mechanical methods for venous thromboembolism prevention. In addition, the appropriate dose, route of administration, duplicity and replacement of the drug were suggested.

**Results:**

We evaluated 9,000 hospitalized medical patients and carried out 77 pharmaceutical interventions. A total of 71 cases (92.21%) adhered to treatment so that non-adherence occurred in 6 cases (7.79%). In 25 cases pharmacologic agents were included and in 20 cases mechanical prophylaxis. Dose adjustments, route, frequency, duplicity and replacement made up 32 cases.

**Conclusion:**

The vertical clinical pharmacist service included the prophylaxis for venous thromboembolism and promotion of appropriate use of medicines in the hospital.

## RESUMO

**Objetivo:**

Descrever as intervenções do serviço do farmacêutico clínico vertical na prevenção do tromboembolismo venoso.

**Métodos:**

Estudo prospectivo, feito em hospital privado. Durante 5 meses, de janeiro a maio de 2012, o farmacêutico clínico avaliou os pacientes clínicos internados sem profilaxia para o tromboembolismo venoso. Se o paciente apresentasse critérios para desenvolver o tromboembolismo e não houvesse contraindicações para a profilaxia, o farmacêutico clínico sugeria a inclusão da profilaxia medicamentosa e/ou mecânica para equipe médica. Além disso, também foram feitas sugestões em relação à dose, via de administração, posologia, duplicidade terapêutica e substituição do medicamento profilático.

**Resultados:**

Foram avaliados 9.000 pacientes internados e feitas 77 intervenções farmacêuticas, houve adesão em 71 casos (92,21%); logo, a não adesão pelo médico ocorreu em 6 casos (7,79%). Em 25 casos houve a inclusão da profilaxia medicamentosa e, em 20 casos, a inclusão da profilaxia mecânica. Os ajustes de dose, via, frequência, duplicidade e substituição somaram 32 casos.

**Conclusão:**

A implantação do serviço do farmacêutico clínico vertical abrangeu a inclusão da profilaxia para tromboembolismo venoso e a promoção do uso correto de medicamentos no ambiente hospitalar.

## INTRODUCTION

Venous thromboembolism (VTE) includes deep vein thrombosis (DVT) and pulmonary thromboembolism (PTE). VTE affects mainly hospitalized patients and its incidence is about one hundred times higher among hospitalized patients than in non-hospitalized patients.^(1)^

Every year in the United States, roughly 200,000 deaths occur due to VTE.^(2)^ VTE is considered a preventable cause of death.^(3)^

Risk of DVT in medical patients is 10-20% and the risk in severe patients is 10-80%.^(4)^

VTE in hospitalized patients could be prevented using pharmacological and/or mechanical measures, which are considered cost-effectiveness.^(5,6)^

VTE prevention was recommended by the Brazilian Medical Association and the Federal Council of Medicine in the Guideline project “venous thromboembolism: prophylaxis in medical patients”, it is also supported by international agencies and institutions such as the American College of Chest Physicians, Joint Commission on Accreditation of Health Care Organizations and The National Quality Forum.^(7-10)^

According to Brazilian guidelines, hospitalized medical patients are at high risk of developing VTE because of several factors.^(7)^

A number of strategies could be used to increase the use of prophylaxis measures in hospitalized patients and, as a consequence, to decrease the risk of VTE development. Professionals in charge to develop such strategies are physicians, nurses and pharmacists. The use of electronic alerts developed by physicians showed decrease of 41% in VET risk.^(11)^ Nurses participations by educational program showed an increase of 16% in number of patients with adequate prophylaxis.^(12)^ However, pharmacists participation in educational program showed an increase from 43 to 58% in the use of VET prophylaxis.^(13)^ Other study showed that use of reminders created by pharmacists increased the use of prophylaxis from 19.5 to 60%.^(14)^

In our hospital, the clinical pharmacist main responsibility is to ensure the appropriate use of medicines. The clinical pharmacist assess medical prescriptions concerning adverse effects, compatibility of injectable drugs, supra or sub-therapeutic doses, allergies, drug interactions, legibility, dilution, route of administration, frequency, drug administration using a probe (concerning risk of obstruction and inadequate absorption), scheduling, adjustments related to kidney function and medicine reconciliation. Besides these activities, clinical pharmacists are also responsible to provide medicines that are not available in the hospital and participate in multidisciplinary visits.^(15)^

Clinical pharmacists could contribute to prevent VET because this professional is part of the multidisciplinary team responsible to provide the best care to inpatients.

## OBJECTIVE

To describe interventions of a vertical clinical pharmacist service in prevention of venous thromboembolism.

## METHODS

This prospective study was carried out at Hospital Israelita Albert Einstein, a tertiary private hospital with around 600 beds. We included only adult medical inpatients admitted from January to May 2012.

At our hospital, there is one clinical pharmacist for each inpatient unit. This pharmacist is responsible for 44 beds on average, with exception of adult intensive care unit that, since March 2012, has 1 pharmacist for each 20 beds.

Since January 2012 a new conception for the role of clinical pharmacist at our institution was created and named vertical clinical pharmacist, *i.e.*, a professional exclusively responsible for VET prevention in the hospital and with the main goal to work along with clinical pharmacists at admission units.

Patients were evaluated by both the vertical clinical pharmacist and the clinical pharmacist at admission units.

Medical patients without anticoagulants were evaluated using an algorithm based on Brazilian Guidelines on Prevention of Venous Thromboembolism in Medical Patients.^(7)^ When a patient without prophylaxis was identified and had risk of developing VET, the clinical pharmacist informed the medical team and suggested prophylaxis. When prophylaxis was accepted, it was included in prescription and registered in the patient's medical record.

The analysis of clinical pharmacist besides indicates prophylaxis also resulted in other interventions, such as: appropriate dose, posology, route of administration, therapeutic duplicity and discussion of best therapeutic option, depend on the patient who was evaluated.

All clinical pharmacists' interventions were gathered monthly, including those cases of non-adherence to prophylaxis by the medical team.

This study was not submitted to Ethical and Research Committee because the analysis included only data from institutional indicators.

## RESULTS

During the 5 months of the study, 77 pharmaceutical interventions were conducted. There were adherence in 71 cases (92.21% - Table 1) and six non-adherence by the medical team (7.79% - Table 2). Upon patients admission at units, the clinical pharmacist and the vertical clinical pharmacist evaluated about 90 patients per day. A total of 9,000 patients were evaluated during the period of the study.

**Table 1:** Monthly clinical pharmacists' interventions that were accepted by medical team

Type of intervention	Jan	Feb	Mar	Apr	May	Total
Inclusion of prophylactic agents	6	2	6	4	7	25
Inclusion of mechanical prophylaxis	4	5	4	7	0	20
Dose adjustment	2	2	6	5	1	16
Posology adjustment	1	0	0	0	1	2
Route of administration adjustment	0	1	1	0	0	2
Changing of prophylactic agent	0	0	2	0	1	3
Suspension of a prescribed prophylactic agent to avoid duplicity	0	0	0	2	1	3
Total	13	10	19	18	11	71

**Table 2:** Monthly interventions suggested by the clinical pharmacist that were not accepted by the medical team

Non-adherence by medical team	Jan	Feb	Mar	Apr	May	Total
Non-acceptance of prophylactic agent	1	0	0	1	1	3
Non-acceptance of mechanical prophylaxis	1	0	0	1	0	2
Non-acceptance of dose adjustment	0	0	1	0	0	1
Total	2	0	1	2	1	6

Prophylactic agent was accepted in 25 cases and mechanical prophylaxis in 20 cases. The use of mechanical prophylaxis was recommended for patients who had contraindications to prophylactic agent, *e.g.*, thrombocytopenia and active bleeding.

There was dose adjustment prescribed in 16 cases. Adjustments occurred mainly in elderly patients with alterations in kidney function. Because of risk of bleeding doses were reduced.

Other interventions were alteration of enoxaparin posology from every 12 hours to once a day (two cases), alteration of route of administration from intravenous to subcutaneous (two cases), replacement of prophylactic medicine because of risk of bleeding in patients with altered kidney function (three cases) and duplicity of therapeutic (three cases).

## DISCUSSION

Several inpatients have risk of VET but often they are not appropriate assessed and neither received appropriate prophylaxis. Data in the literature show this absence of assessment or prophylaxis.^(16,17)^ This study confirm this absence especially because pharmacist included prophylaxis in 45 cases.

No studies on participation of pharmacist in VET prophylaxis was conducted Brazil, but some international experiences shown good results concerning increase of number of patients with prophylaxis by intervention done by the clinical pharmacist both by participation in multidisciplinary visits or by active seeking.^(14,18)^

Prophylactic agents are indicated injectable anticoagulant drugs such as non-fractionated heparin, heparin of low molecular weight, fondaparinux and oral medicines as factor Xa and thrombin inhibitors. Patients who had contraindication to anticoagulants, the mechanical prophylaxis (gradual compression stockings and the use of intermittent pneumatic compression devices) is recommended.^(3,6)^ Of our sample, 22 cases had contraindications for prophylactic agents. For these patients the mechanical prophylaxis was suggested.

Our findings showed that pharmacists contributed to improve the use of prophylaxis when identified and suggested its use, in addition they promoted the appropriate use of medicines. Pharmacists' suggestions were accepted in 92.21% of cases, which were similar to pharmacists' interventions in the hospital.^(15)^

This study has two limitations. Firstly, is the lack of data on patients who developed VET during hospitalization, which did not enabled to demonstrate the impact of role of pharmacists concerning to the number of VET patients. Secondly, there was a difficult to evaluate patients in the appropriate manner on prophylaxis because VET is multifactorial.

We found patients with high risk of VET without prophylaxis, but the medical team recorded their decision of non-adherence to prophylaxis in the patient's medical record. In such cases, the pharmacist did not interfere.

## CONCLUSION

It is important that hospitals adopt strategies to decrease chances of inpatients to develop venous thromboembolism. The consequences of VET could be fatal, besides the likelihood to increase length of hospital stay and associated direct and indirect costs.

The implementation of vertical clinical pharmacist service included the venous thromboembolism prophylaxis and the promotion of appropriate use of medicines in the hospital environment.

Clinical pharmacists' interventions were the inclusion of prophylactic agents, inclusion of mechanical prophylaxis, dose adjustment, posology adjustment, route of administration adjustment, replacement of prophylactic medicines and suspension of prophylaxis to avoid therapeutic duplicity.
